# Prevalence and risk factors of *Helicobacter pylori* infection in urban children aged 6–13 years in Fuzhou, China: a cross-sectional study in a high gastric cancer incidence region

**DOI:** 10.3389/fped.2025.1457195

**Published:** 2025-07-16

**Authors:** Youtao Chen, Xiaoting Pan, Yanyan Wang, Longzhen Lin, Xiaoyan Chen, Yan Chen, Hong Ye

**Affiliations:** ^1^ Department of Gastroenterology and Nutritional Medicine, Fujian Children's Hospital (Fujian Branch of Shanghai Children's Medical Center), Fuzhou, Fujian, China; ^2^ College of Clinical Medicine for Obstetrics & Gynecology and Pediatrics, Fujian Medical University, Fuzhou, Fujian, China; ^3^ Department of Pediatrics, Fujian Maternity and Child Health Hospital, Fuzhou, Fujian, China; ^4^ Department of Pediatrics, The People’s Hospital of Changle District, Fuzhou, Fujian, China

**Keywords:** *Helicobacter pylori*, China, children, prevalence, 13C-urea breath test

## Abstract

**Background:**

The burden of *Helicobacter pylori* infection remains a global public health concern, particularly due to its lifelong risk of gastric cancer starting from childhood. Comprehensive data on the prevalence of *H. pylori* infection and associated risk factors among children are limited. This study aimed to investigate the prevalence and risk factors of *H. pylori* infection among children from Fuzhou, a high gastric cancer incidence region in southeastern China.

**Methods:**

In this 2023 cross-sectional study, urban children aged 6–13 years were enrolled via stratified random sampling. Ethical approval was obtained prior to the study. Diagnosis was performed using the ¹³C-urea breath test, and risk factors were assessed through structured questionnaires covering demographics, lifestyle, diet, household conditions, and socioeconomic status.

**Results:**

A total of 338 children were included. The overall prevalence of *H. pylori* infection was 22.2% (95% CI: 0.178–0.266), with age-specific rates increasing from 8.3% (6–7 years) to 34.7% (10–11 years). Multivariate logistic regression analysis identified frequent desserts/fried foods consumption as a risk factor (OR = 2.02, 95% CI: 1.13–3.63, *p* = 0.018), while higher annual household income was protective (OR = 0.567, 95% CI: 0.32–1.00, *p* = 0.048).

**Conclusions:**

The age-dependent increase in *H. pylori* infection suggests cumulative environmental exposure. Modifiable risk factors (dietary habits, household crowding) and protective factors (income, hygiene practices) highlight the need for targeted public health interventions for pediatric populations at high risk.

## 1 Introduction

*Helicobacter pylori* (*H. pylori*), first detected in 1983, has been recognized as a major cause of gastritis and peptic ulcer disease in adults and children, and as a significant risk factor for gastric cancer (1). Despite extensive research, the origin and transmission route of *H. pylori* infections remain uncertain. Previous studies suggest that potential transmission may occur via oral-oral, gastro-oral, or fecal-oral routes (1, 2). Globally, *H. pylori* infection exhibits high prevalence among children and is predominantly acquired during childhood, with significant familial clustering. Research has established that the primary transmission routes involve close interpersonal contact, specifically via the oral-oral route (e.g., sharing utensils, pre-masticated feeding by caregivers, salivary contact) and the fecal-oral route (e.g., ingestion of contaminated water or food), while zoonotic or vector-borne transmission cannot be entirely ruled out. Consequently, the risk of *H. pylori* acquisition during childhood is profoundly influenced by multiple factors, including socioeconomic status, household hygiene practices, dietary habits (such as consumption of raw foods, street food safety, drinking water safety), lifestyle, and environmental factors (e.g., water quality) (3–5). Numerous studies have demonstrated that the prevalence of *H. pylori* infection varies according to age, geographic area, sanitary conditions during childhood, and socioeconomic status (3, 4).

*H. pylori* infection is predicted to affect approximately 50% of the world's population (5). The global prevalence among children was estimated at 33% (6); however, this estimate lacks regional, national, or geographical breakdown-data essential for targeted preventive measures. Geographic variation in prevalence is significant. In high-income countries, declining infection rates correlate with improved living standards, while most developing nations maintain high prevalence. Notably, prevalence among children in low­income and middle­income countries reaches nearly twice that in high­income countries (43.2% vs. 21.7%) (4).

In China, *H. pylori* infection accounts for 9.2% and 9.8% of all cancer cases and deaths, respectively (7), primarily due to gastric cancer (8, 9). A 2022 meta-analysis on infection prevalence among Chinese children reported an overall infection rate of 30.31% (10). However, China's nationwide prevalence remains incompletely characterized over recent decades. Furthermore, infection rates among children vary significantly across regions and between urban-rural settings with different socioeconomic levels. Given this substantial disease burden and associated gastric cancer risk, population-based screening represents a key prevention strategy.

Fuzhou, a southeastern China city and the core city of the West Coast Economic Zone, features a developed economy, a highly mobile population with diverse origins, and consequently, an exceptionally diverse culinary culture and lifestyle. This unique context directly impacts the risk profiles for *H. pylori* transmission: the oral-oral route may be complicated by heterogeneous caregiving practices and varying levels of intimate contact within families, particularly those originating from regions with high *H. pylori* prevalence; the fecal-oral route poses heightened uncertainty due to potential variations in water safety, sanitation infrastructure disparities (especially in urban-rural fringes), and the highly variable hygiene standards of street food vendors near schools, which cater to diverse regional tastes. Given that *H. pylori* is classified by the World Health Organization (WHO) as a Group 1 carcinogen and persistent infection is a key risk factor for gastric carcinogenesis, prevention during childhood—the primary window for acquisition—is critically important (1). Yet Fuzhou, despite being a high-risk area for gastric cancer, currently lacks epidemiological data on *H. pylori* infection in children, which may limit the translation of this knowledge into public health interventions for *H. pylori* eradication and gastric cancer prevention. To better understand the transmission dynamics and population health impact of *H. pylori*, greater research focus should be directed toward early childhood infection, as accumulating evidence suggests that *H. pylori* acquisition occurs predominantly during this period (11).

Several methods have been developed to detect *H. pylori* infection. Currently accessible diagnostic tests include both invasive and non-invasive methods. Non-invasive methods comprise the urea breath test (UBT), stool antigen test, and serological assays, while invasive techniques include endoscopy with histopathological examination, rapid urease test, culture, and polymerase chain reaction (12). The ^13^C-urea breath test (^13^C-UBT) is particularly sensitive and specific for pediatric diagnosis, especially when endoscopy is not routinely performed (13, 14). In children aged 6 years and older, its sensitivity and specificity may exceed 97.7% and 96.6%, respectivel (15). Our study used the ^13^C-UBT to determine the *H. pylori* prevalence in children.

Therefore, this study aimed to investigate the prevalence of *H. pylori* infection using the ^13^C-UBT in a randomly selected population of school-aged children from Fuzhou, China, and to examine potential risk actors influencing *H. pylori* acquisition.

## 2 Methods

### 2.1 Subjects

This cross-sectional study was conducted in 2023 at a randomly selected primary school in Fuzhou,southeastern China. School-aged children (6–13 years) were invited to participate using whole-cluster sampling. Volunteers were screened against inclusion criteria. A stratified random sample was then selected based on age at last birthday (stratified into 6–7, 8–9, 10–11, and 12–13 years). Within each stratum, children were randomly selected using computer-generated numbers proportional to stratum size. *H. pylori* infection status was determined by ¹³C-urea breath test (¹³C-UBT). All selected participants provided valid ¹³C-UBT results and completed study questionnaire. Written informed consent was obtained from parents/guardians after detailed study explanation. The study was approved by the Institutional Review Board.

### 2.2 Exclusion criteria

The following individuals were excluded from our study: children taking medications such as antibiotics, H2 receptor blockers, iron, proton pump inhibitors, etc. in the last one month; children with acute gastroenteritis with severe vomiting and diarrhea; children with other congenital gastrointestinal disorders.

### 2.3 Questionnaires

A questionnaire was administered requesting information on: age, gender, personal dietary habits, annual household income, household crowding (defined as total occupants per dwelling), drinking water sources, and pet ownership.

### 2.4 13C urea breath test

In children, the 13C urea breath test (UBT) is a widely used test to detect *H. pylori* infection with a diagnostic accuracy of >95% (16). UBT is widely used because breath samples are easy to collect and can even be sent by mail to a central laboratory for analysis. The 13C UBT was performed after 2 h of fasting. After collecting the initial breath sample, the children were given 80–100 ml of warm drinking water containing 50 mg 13C-urea. A second breath sample was collected 30 min later. The samples were analyzed by infrared spectrometer. The results were considered positive when delta over baseline (DOB) was 4.0 per thousand (‰). The breath test using infrared spectroscopy has been validated in children. The test has proven to be highly sensitive and specific for the diagnosis of the infection in children of all ages, especially those aged 5 years and older (17).

### 2.5 Data analysis

All data were entered into the Statistical Package for the Statistical Product and Service Solutions (SPSS) statical software, for statistical analysis. The results of the questionnaire are expressed as frequency distributions and were calculated in percentages. Associations between the outcome measure (*H. pylori* infection) and various predictors were assessed using Pearson's chi-squared test. A level of *p* < 0.05 was considered statistically significant for all analyses. These predictor variables were then considered for inclusion in multivariate logistic regression models to assess their independent effects. Multiple logistic regression was used to identify independent predictors of *H. pylori* infection in children as dependent variable. All variables with a *p*-value of 0.05 or less were included in the full logistic regression model.

## 3 Results

### 3.1 Demographic information

The demographic characteristics of the participants are listed in Table 1. Of 1,246 school-aged children (6–13 years) in the selected school, 883 volunteered to participate. After screening, 861 children met inclusion criteria. A stratified random sample of 338 children was selected (proportional to age strata: 6–7, 8–9, 10–11, and 12–13 years). All enrolled participants provided valid ¹³C-UBT results and completed questionnaires. The cohort consisted of 155 girls (45.9%) and 183 boys (54.1%), with ages ranging from 6 to 13 years (mean age 9.95 ± 1.81 years). Age distribution was as follows: 48 children (14.2%) in the 6–7-year-old group, 130 (38.5%) in the 8–9-year-old group, 95 (28.1%) in the 10–11-year-old group, and 65 (19.2%) in the ≥12-year-old group.

**Table 1:** Demographic characteristics and Helicobacter pylori infection Status of enrolled children in Fuzhou, China (*N* = 338), stratified by gender and Age groups.

Variable	Number infected (%)	Not infected (%)	Total	*p* value
Participants	75 (22.2)	263 (77.8)	338	
Gender				
Male	43 (23.5)	140 (76.5%)	183 (54.1%)	0.529
Female	32 (20.6)	123 (79.4%)	155 (45.9%)	
Age (years)				0.002
6–7	4 (8.3)	44 (91.7)	48	
8–9	25 (19.2)	105 (80.8)	130	
10–11	33 (34.7)	62 (65.3)	95	
12–13	13 (20.0)	52 (80.0)	65	

**Figure 1:**
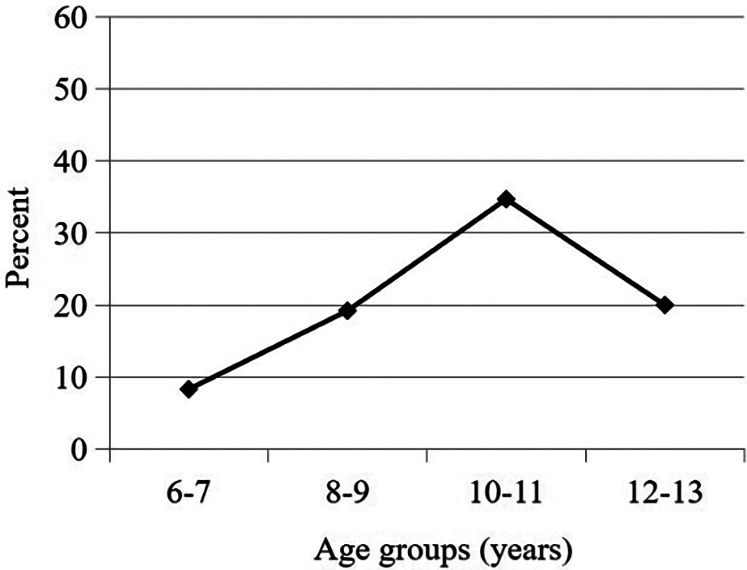
Age distribution of *H. pylori* infection.

The overall prevalence of *H. pylori* infection was 22.2% (75/338) (95% CI: 0.0178–0.266). There was no statistical difference between the average prevalence of the male gender (23.5%; 43/183) and the female gender (20.6%; 32/155) (*p* = 0.529). The age specific prevalence of *H. pylori* infection is presented in Figure 1. *H. pylori* infection was most common for those children of 10–11 years of age, in whom 34.7% (33/95) of all children were positive. The age group with the least *H. pylori* infection rate was 6–7 years with prevalence of 8.3% (4/48).

### 3.2 Risk factors of *H. pylori* infection

The Pearson's chi-square test for qualitative data was performed separately for factors associated with *H. pylori* infection and personal dietary habits. Table 2 stratifies the *H. pylori* infection status of the participants with regards to personal dietary habits. A statistically significant difference was found in the prevalence of *H. pylori* infection of the children and Personal dietary habits, including eat raw vegetable (*c*^2^ = 4.380, *p* < 0.05), eat dessert/fried foods (*c*^2^ = 3.952, *p* < 0.05), eat fast food frequently (*c*^2^ = 6.542, *p* < 0.05) and daily salt intake (*c*^2^ = 4.699, *p* < 0.05).

**Table 2:** Univariate analysis of dietary habit associations with Helicobacter pylori infection status in children from Fuzhou, China.

Variable	Number infected (%)	Number not infected (%)	*χ*²	*p* value
Raw vegetable ingestion			4.380	0.036
Yes	41 (27.5)	108 (72.5)		
No	34 (18.0)	155 (82.0)		
Eat dessert/fried foods frequently			3.952	0.047
Yes	32 (28.6)	80 (71.4)		
No	43 (19.0)	183 (81.0)		
Eat fast food frequently			6.542	0.011
Yes	7 (50.0)	7 (50.0)		
No	68 (21.0)	256 (79.0)		
Daily salt intake (g/day)			4.699	0.030
<6 g/day	54 (26.1)	153 (73.9)		
≥6 g/day	21 (16.0)	110 (84.0)		
Eat fruit frequently (≥3 times/week)			0.635	0.425
Yes	57 (21.3)	211 (78.7)		
No	18 (25.7)	52 (74.3)		
Often eat at school (≥3 times/week)			0.148	0.700
Yes	50 (22.8)	169 (77.2)		
No	25 (21.0)	94 (79.0)		
Wash/peel before eating fruit			0.176	0.675
Yes	7 (19.4)	29 (80.6)		
No	68 (22.5)	234 (77.5)		
Source of drinking water			1.349	0.245
Well water	13 (28.9)	32 (71.1)	45	
Municipal water	62 (21.2)	231 (78.8)	293	

About 44.1% of the participants eat raw vegetable, whereas 27.5% (41/149) of those eating raw vegetable were *H. pylori* positive, only 18% (34/189) of those do not eat raw vegetable were *H. pylori* positive. While eating fruit frequently, eating at school, wash or peel before eating fruit and source of drinking water were not associated with the infection (*p* > 0.05). It was not possible to evaluate the association between frequency of *H. pylori* infection and frequency of eating fast food because 96% of the participants do not eat fast food frequently.

Table 3 stratifies the study participants with regards to *H. pylori* infection and its association with educational level of parents and household size. The results showed that family year income, participant household size and frequency of Household indoor cleaning was significantly associated with the prevalence of *H. pylori* infection (*p* < 0.05, Pearson's chi-squared test). However, the education level of family members caring for children and household pets were not significantly associated with the infection rate.

**Table 3:** Multivariate regression analysis of socioeconomic factors associated with Helicobacter pylori infection in children: parental education and household density in Fuzhou, China.

Variable	Number Infected (%)	Number not infected (%)	Total	*p* value
Annual household income (yuan/year)				0.039
<100,000	37 (28.0)	95 (72.0)	132	
≥100,000	38 (18.4)	168 (81.6)	206	
Number of people in household				0.04
<3	9 (13.0)	60 (87.0)	69	
≥3	66 (24.5)	203 (75.5)	269	
Frequency of Household indoor cleaning (times/week)				0.027
<4	16 (34.8)	30 (65.2)	46	
≥4	59 (20.2)	233 (79.8)	292	
Education level of family members caring for children				0.855
Above high school	13 (21.3)	48 (78.7)	61	
Below high school	62 (22.4)	215 (77.6)	277	
Domestic animal				0.814
Yes	9 (23.7)	29 (76.3)	38	
No	66 (22.0)	234 (78.0)	300	

All the predictor variables found to be significant from the crude analyses were then investigated to assess their independent effects by introducing them into multivariate logistic regression models. The results are listed in Table 4 which showed that eat dessert or fried foods frequently (OR = 2.02, 95% CI: 1.13–3.63, *p* = 0.018) and larger household size (≥4) (OR = 2.749, 95% CI: 1.19–6.34, *p* = 0.018) were risk factors for *H. pylori* infection, while higher family year income (OR = 0.567, 95% CI: 0.32–1.00, *p* = 0.048) and frequency of household indoor cleaning (OR = 0.323, 95% CI: 0.15–0.7, *p* = 0.004) were protective factors for *H. pylori* infection.

**Table 4:** Univariate prevalence rates and multivariate adjusted odds ratios of Helicobacter pylori infection associated with Key socioeconomic and dietary risk factors in children from Fuzhou, China.

Variable	OR	95% CI	*p* value
Eat dessert or fried foods frequently	2.02	1.13–3.63	0.018
Annual household income	0.567	0.32–1.00	0.048
Frequency of Household indoor cleaning	0.323	0.15–0.7	0.004
Number of people in household	2.749	1.19–6.34	0.018

## 4 Discussion

### 4.1 Epidemiological characteristics of *H. pylori* infection

*H. pylori* infection affects more than half of the world's population, including children. *H. pylori* is the only bacterium recognized by WHO as a level 1 carcinogen (18), and persistent colonization along with the resulting chronic inflammation are key factors in the development of gastric malignant tumors (5, 19). Given its predominant fecal-oral transmission during childhood (20, 21). Home and school environments, as well as socioeconomic and hygienic conditions during childhood, may be important determinants of *H. pylori* transmission.Fuzhou, a city in southeastern China, is a high-risk area for gastric cancer. In 2010, the gastric cancer mortality rate in Changle District of Fuzhou was 3.78 per 100, 000 (22); yet the region lacked prior epidemiological data on urban school-aged children—a gap this study addresses.

### 4.2 Regional prevalence patterns and study significance

The reported prevalence of *H. pylori* infection in adults varies from 24% to 73% across populations, depending on geographical region and economic development, with an estimated global prevalence of approximately 50% (23). However, the epidemiology of *H. pylori* infection in children exhibits marked heterogeneity compared with adults. Many infected children are asymptomatic, and only 5% develop peptic ulcer disease (24). Notably, the highest global prevalence occurs in Africa (44.1%) and the lowest in the Western Pacific region according to Changzheng Yuan et al. (4). Although longitudinal studies report varying rates, most consistently show pediatric prevalence around 30% globally (6), reflecting socioeconomic gradients—with developed regions demonstrating significantly lower rates due to improved sanitation and living standards.

In China, a 2021 meta-analysis revealed an overall pediatric/adolescent infection rate of 28.0% (25), while a 2022 study highlighted striking regional disparities: Northwest China (40.09%), Central-South (34.78%), East China (31.38%), Northeast (24.47%), North (17.34%), and Southwest (15.18%) (10). Against this backdrop, our finding of 22.2% prevalence in Fuzhou (within East China) suggests that coastal metropolitan advantages may further reduce transmission risk—providing critical baseline data for high-risk area interventions.

### 4.3 Mechanistic insights into risk factors

Our study demonstrated no statistical difference between the prevalence in boys (23.5%) and girls (20.6%), consistent with Yuan et al.'s global pediatric report (4). This contrasts with adult studies showing male predominance [e.g., De Martel et al. (26)], which meta-analyses attribute to behavioral divergence post-adolescence (27). The absence of gender disparity in school-aged children likely stems from socio-behavioral homogenization during pre-adolescent development: cultural practices such as communal dining and shared toy usage equalize exposure risk through frequent interpersonal contact; undifferentiated hygiene behaviors manifest as similar handwashing frequency and sanitation awareness across genders; and limited gender-role segregation prior to puberty minimizes divergence in play activities and social interactions that could modulate transmission pathways.

Consistent with global patterns of childhood acquisition (2), our data demonstrated a significant age-gradient in *H. pylori* prevalence, increasing from 8.3% (6–7 years) to 34.7% (10–11 years). This progression underscores early childhood as the critical window for pathogen colonization, emphasizing the importance of targeted prevention in primary education to mitigate long-term gastric cancer risk.

This study analyzed the impact of dietary habits on *H. pylori* infection in school-age children, as diet may influence the occurrence and development of infection. The results showed that consumption of raw vegetables, frequent consumption of desserts/fried foods, frequent consumption of fast foods, and daily salt intake were significantly associated with *H. pylori* infections in school-age children. Multifactorial regression identified frequent consumption of desserts/fried foods as an independent risk factor (OR = 2.02, 95% CI: 1.13–3.63, *p* = 0.018). The pathophysiological basis of this association likely involves a synergistic triad of mechanisms: high-fat diets induce duodenogastric reflux of bile acids that disrupt gastric mucin barrier integrity, while thermal processing of fried foods generates dietary carcinogens such as acrylamide that promote epithelial inflammation and oxidative stress (28). Regarding environmental transmission, while water represents a potential source supported by multiple studies (29, 30), our findings align with evidence indicating *H. pylori* rapidly loses viability in aquatic environments (31): Pearson's chi-squared test showed no significant difference in drinking water sources between infected and uninfected children (*p* > 0.05).

Educational status is used as a proxy marker for socioeconomic status and a critical determinant of *H. pylori* prevalence in both developed and resource-limited settings (32, 33). In this study, univariate analysis results indicated that annual household income, number of household members, and frequency of household indoor sanitation were all significantly associated with *H. pylori* infection status; parental educational status did not significantly affect prevalence. Following multifactorial regression analysis, the number of family members was identified as a risk factor, while higher annual househoud income and increased frequency of household indoor cleaning were protective factors against *H. pylori.* Household crowding has been established as a risk factor. Within our social context, poverty and lower socioeconomic status correlate with larger household size. Therefore, considering the transmission mode, the observed increase in prevalence with larger household numbers is consistent with expectations. The results demonstrated no association between pet ownership and *H. pylori* infection in children, suggesting transmission may not occur through pets, though further research is needed. Consequently, proper hand hygiene and effective waste management systems can help control household infections, particularly in crowded settings. Additionally, ensuring thorough washing and cooking of vegetables given to children, promoting healthy dietary habits (including reduced consumption of desserts and fried foods), and encouraging low-salt diets are important preventive measures against childhood *H. pylori* infections.

### 4.4 Public health implications and limitations

Although this cross-sectional study provides foundational evidence for *H. pylori* prevention while acknowledging inherent causal inference limitations, its crucial identification of modifiable dietary and household transmission risks directly informs targeted interventions. These discoveries justify three public health priorities: implementing annual screening in elementary schools, regulating fried food availability in educational settings, and providing hygiene subsidies for crowded households. To overcome current constraints—particularly limited generalizability beyond urban Chinese cohorts, recall bias in self-reported dietary data, and unverified transmission mechanisms—future research must expand rural/urban cohort diversity through multicenter sampling, utilize objective biomarkers such as urinary nitrate to quantify dietary exposure, and validate transmission pathways through longitudinal studies integrating strain genotyping.

## 5 Conclusions

In summary, this study shows that the overall prevalence of *H. pylori* infection was 22.2% in children living in Fuzhou, a city with high prevalence of gastric cancer in southeastern China. It identifies the age, frequent consumption of desserts/fried foods, and larger household size as risk factors for the infection, while higher annual household income and more frequent household indoor cleaning were protective factors. Parental educational status, drinking water source, and pet ownership did not affect *H. pylori* prevalence. The possible modes of transmission of *H. pylori* among young children remain to be investigated.

These findings highlight the need for targeted nutritional education programs discouraging frequent dessert/fried food consumption in children. Hygiene interventions promoting regular indoor cleaning could be prioritized, especially in larger households.Socioeconomically vulnerable groups (e.g., lower-income families) should be prioritized for screening and prevention efforts, given the income-protection association.

Mechanistic studies are urgently needed to clarify early childhood transmission routes, particularly since water sources/animal contact showed no effect in this study. Longitudinal studies should validate whether reducing fried food/dessert intake lowers infection rates. Cost-effectiveness analyses of hygiene campaigns in high-risk regions like Fuzhou are recommended to guide policy.

## Data Availability

The raw data supporting the conclusions of this article will be made available by the authors, without undue reservation.

## References

[1] Marginean CM Cioboata R Olteanu M Vasile CM Popescu M Popescu AIS The importance of accurate early diagnosis and eradication in Helicobacter pylori infection: pictorial summary review in children and adults. Antibiotics. (2022) 12(1):60. 10.3390/antibiotics12010060 36671261 PMC9854763

[2] Zamani M Ebrahimtabar F Zamani V Miller WH Alizadeh-Navaei R Shokri-Shirvani J Systematic review with meta-analysis: the worldwide prevalence of Helicobacter pylori infection. Aliment Pharmacol Ther. (2018) 47(7):868–76. 10.1111/apt.14561 29430669

[3] Peleteiro B Bastos A Ferro A Lunet N. Prevalence of Helicobacter pylori infection worldwide: a systematic review of studies with national coverage. Dig Dis Sci. (2014) 59(8):1698–709. 10.1007/s10620-014-3063-0 24563236

[4] Yuan C Adeloye D Luk TT Huang L He Y Xu Y The global prevalence of and factors associated with Helicobacter pylori infection in children: a systematic review and meta-analysis. Lancet Child Adolesc Health. (2022) 6(3):185–94. 10.1016/S2352-4642(21)00400-4 35085494

[5] Koletzko S Jones NL Goodman KJ Gold B Rowland M Cadranel S Evidence-based guidelines from ESPGHAN and NASPGHAN for Helicobacter pylori infection in children. J Pediatr Gastroenterol Nutr. (2011) 53(2):230–43. 10.1097/MPG.0b013e3182227e90 21558964

[6] Zabala Torrres B Lucero Y Lagomarcino AJ Orellana-Manzano A George S Torres JP Review: prevalence and dynamics of Helicobacter pylori infection during childhood. Helicobacter. (2017) 22(5):e12399. 10.1111/hel.12399 28643393

[7] Xiang W Shi J-F Li P Wang J-B Xu L-N Wei W-Q Estimation of cancer cases and deaths attributable to infection in China. Cancer Causes Control. (2011) 22(8):1153–61. 10.1007/s10552-011-9791-y 21667067

[8] Jing JJ Liu HY Hao JK Wang LN Wang YP Sun LH Gastric cancer incidence and mortality in Zhuanghe, China, between 2005 and 2010. World J Gastroenterol. (2012) 18(11):1262–9. 10.3748/wjg.v18.i11.1262 22468091 PMC3309917

[9] Yang L. Incidence and mortality of gastric cancer in China. World J Gastroenterol. (2006) 12(1):17–20. 10.3748/wjg.v12.i1.17 16440411 PMC4077485

[10] Wenhong L Ziwei L Na W Jiaxiang Y. Prevalence of Helicobacter pylori infection and associated risk factors among Chinese children: a meta-analysis. Chin Gen Pract. (2022) 22(28):3569–78. 10.12114/j.issn.1007-9572.2022.0028

[11] Salama NR Hartung ML Müller A. Life in the human stomach: persistence strategies of the bacterial pathogen Helicobacter pylori. Nat Rev Microbiol. (2013) 11(6):385–99. 10.1038/nrmicro3016 23652324 PMC3733401

[12] Sun Q Yuan C Zhou S Lu J Zeng M Cai X Helicobacter pylori infection: a dynamic process from diagnosis to treatment. Front Cell Infect Microbiol. (2023) 13:1257817. 10.3389/fcimb.2023.1257817 37928189 PMC10621068

[13] Cardos AI Maghiar A Zaha DC Pop O Fritea L Miere (Groza) F Evolution of diagnostic methods for Helicobacter pylori infections: from traditional tests to high technology, advanced sensitivity and discrimination tools. Diagnostics. (2022) 12(2):508. 10.3390/diagnostics12020508 35204598 PMC8871415

[14] Graham DY Miftahussurur M. Helicobacter pylori urease for diagnosis of Helicobacter pylori infection: a mini review. J Adv Res. (2018) 13:51–7. 10.1016/j.jare.2018.01.006 30094082 PMC6077137

[15] Leal YA Flores LL Fuentes-Pananá EM Cedillo-Rivera R Torres J. 13C-urea Breath test for the diagnosis of Helicobacter pylori infection in children: a systematic review and meta-analysis. Helicobacter. (2011) 16(4):327–37. 10.1111/j.1523-5378.2011.00863.x 21762274

[16] Braden B. Diagnosis of Helicobacter pylori infection. Praxis. (2012) 101(12):787–92. 10.1024/1661-8157/a000985 22669782

[17] Czinn SJ. Helicobacter pylori infection: detection, investigation, and management. J Pediatr. (2005) 146(3):S21–6. 10.1016/j.jpeds.2004.11.037 15758899

[18] de Martel C Georges D Bray F Ferlay J Clifford GM. Global burden of cancer attributable to infections in 2018: a worldwide incidence analysis. Lancet Glob Health. (2020) 8(2):e180–e90. 10.1016/S2214-109X(19)30488-7 31862245

[19] Kaur CP Vadivelu J Chandramathi S. Impact of Klebsiella pneumoniae in lower gastrointestinal tract diseases. J Dig Dis. (2018) 19(5):262–71. 10.1111/1751-2980.12595 29573336

[20] Ding S-Z Du Y-Q Lu H Wang W-H Cheng H Chen S-Y Chinese consensus report on family-based Helicobacter pylori infection control and management (2021 edition). Gut. (2022) 71(2):238–53. 10.1136/gutjnl-2021-325630 34836916 PMC8762011

[21] Ding SZ. Global whole family based-Helicobacter pylori eradication strategy to prevent its related diseases and gastric cancer. World J Gastroenterol. (2020) 26(10):995–1004. 10.3748/wjg.v26.i10.995 32205991 PMC7080999

[22] Tie-hui C Shao-fen H Xiao-Qing L Xiu-quan L Wen-ling Z Shu-guang L. Epidemiological characteristics and trend of death among pationts with malignant tumor in Fujian from 2007 to 2011. Chin Prev Med. (2013) 14(5):370–4. 10.16506/j.1009-6639.2013.05.020

[23] Hooi JKY Lai WY Ng WK Suen MMY Underwood FE Tanyingoh D Global prevalence of Helicobacter pylori infection: systematic review and meta-analysis. Gastroenterology. (2017) 153(2):420–9. 10.1053/j.gastro.2017.04.022 28456631

[24] Poddar U. Helicobacter pylori: a perspective in low- and middle-income countries. Paediatr Int Child Health. (2019) 39(1):13–7. 10.1080/20469047.2018.1490100 29987976

[25] Ren S Cai P Liu Y Wang T Zhang Y Li Q Prevalence of Helicobacter pylori infection in China: a systematic review and meta-analysis. Gastroenterol Hepatol. (2022) 37(3):464–70. 10.1111/jgh.15751 34862656

[26] De Martel C Parsonnet J. Helicobacter pylori infection and gender: a meta-analysis of population-based prevalence surveys. Dig Dis Sci. (2006) 51(12):2292–301. 10.1007/s10620-006-9210-5 17089189

[27] Ibrahim A Morais S Ferro A Lunet N Peleteiro B. Sex-differences in the prevalence of Helicobacter pylori infection in pediatric and adult populations: systematic review and meta-analysis of 244 studies. Dig Liver Dis. (2017) 49(7):742–9. 10.1016/j.dld.2017.03.019 28495503

[28] Prashar A Capurro MI Jones NL. Under the radar: strategies used by Helicobacter pylori to evade host responses. Annu Rev Physiol. (2022) 84:485–506. 10.1146/annurev-physiol-061121-035930 34672717

[29] Vesga F Moreno Y Ferrús MA Ledesma-Gaitan LM Campos C Trespalacios AA. Correlation among fecal indicator bacteria and physicochemical parameters with the presence of Helicobacter pylori DNA in raw and drinking water from Bogotá, Colombia. Helicobacter. (2019) 24(3):e12582. 10.1111/hel.12582 30950129

[30] Castillo M Bernabe L Castaneda CA Chavez I Ruiz E Barreda F Helicobacter Pylori detected in tap water of Peruvian patients with gastric cancer. Asian Pac J Cancer Prev. (2019) 20(11):3193–6. 10.31557/APJCP.2019.20.11.3193 31759341 PMC7062988

[31] Inamasu Y Ogawa M Saito M Harada M Fukuda K. Helicobacter pylori results in lysis and death after exposure to water. Helicobacter. (2022) 27(5):e12921. 10.1111/hel.12921 36089840

[32] Jafri W Yakoob J Abid S Siddiqui S Awan S Nizami SQ. Helicobacter pylori infection in children: population-based age-specific prevalence and risk factors in a developing country. Acta Paediatr. (2010) 99(2):279–82. 10.1111/j.1651-2227.2009.01542.x 19839955

[33] Al-Hussaini A Al Jurayyan A Bashir S Alshahrani D. Where are we today with Helicobacter pylori infection among healthy children in Saudi Arabia?. Saudi J Gastroenterol. (2019) 25(5):309–18. 10.4103/sjg.SJG_531_18 31006713 PMC6784433

